# Effect of Work-to-Rest Cycles on Cardiovascular Strain and Maximal Oxygen Uptake during Heat Stress

**DOI:** 10.3390/ijerph20054580

**Published:** 2023-03-04

**Authors:** Anne M. Mulholland, Hillary A. Yoder, Jonathan E. Wingo

**Affiliations:** 1 Department of Kinesiology, The University of Alabama, Tuscaloosa, AL 35487, USA; 2 Department of Kinesiology, New Mexico State University, Las Cruces, NM 88003, USA

**Keywords:** cardiovascular drift, occupational health and safety, work:rest ratios, work capacity

## Abstract

Cardiovascular drift—a progressive increase in heart rate (HR) and decrease in stroke volume (SV) during prolonged exercise—is exacerbated by heat stress and thermal strain, and often accompanied by a decrease in work capacity (indexed as maximal oxygen uptake [$\dot{V}$O_2max_]). To attenuate physiological strain during work in the heat, use of work:rest ratios is recommended by the National Institute for Occupational Safety and Health. The purpose of this study was to test the hypothesis that during moderate work in hot conditions, utilizing the recommended 45:15 min work:rest ratio would result in cardiovascular drift ‘accumulating’ over consecutive work:rest cycles and accompanying decrements in $\dot{V}$O_2max_. Eight people (5 women; (mean ± SD) age = 25 ± 5 y; body mass = 74.8 ± 11.6 kg; $\dot{V}$O_2max_ = 42.9 ± 5.6 mL·kg^−1^·min^−1^) performed 120 min of simulated moderate work (201–300 kcal·h^−1^) in hot conditions (indoor wet-bulb globe temperature = 29.0 ± 0.6 °C). Participants completed two 45:15 min work:rest cycles. Cardiovascular drift was evaluated at 15 and 45 min of each work bout; $\dot{V}$O_2max_ was measured after 120 min. On a separate day, $\dot{V}$O_2max_ was measured after 15 min under identical conditions for comparison before and after cardiovascular drift occurred. HR increased 16.7% (18 ± 9 beats·min^−1^, *p* = 0.004) and SV decreased 16.9% (−12.3 ± 5.9 mL, *p* = 0.003) between 15 and 105 min, but $\dot{V}$O_2max_ was unaffected after 120 min (*p* = 0.14). Core body temperature increased 0.5 ± 0.2 °C (*p* = 0.006) over 2 h. Recommended work:rest ratios preserved work capacity but did not prevent the accumulation of cardiovascular and thermal strain.

## 1. Introduction

Self-paced work in hot conditions results in diminished productivity [1], which is partly attributable to behavioral thermoregulation, a self-preservation process where pace slows in response to the perception of heat stress [2]. Decreased productivity during heat stress can also be impacted by hyperthermia, dehydration, fatigue, and cardiovascular strain [3,4,5]. Understanding heat stress limitations on worker productivity is important because preserving productivity for economic reasons may be at odds with occupational health and safety regulations [6].

The magnitude of cardiovascular strain workers experience under heat stress can be represented as cardiovascular drift, a well-established phenomenon that occurs during continuous, submaximal, steady-state exercise, and is characterized by a progressive increase in heart rate (HR) and decrease in stroke volume (SV) over time. The drift in HR and SV is often concomitant to a decrease in work capacity, indexed as maximal oxygen uptake ($\dot{V}$O_2max_) [4]. Heat stress and hyperthermia amplify cardiovascular drift and accompanying decrements in $\dot{V}$O_2max_ in as little as 45 min during exercise [4,7]. As cardiovascular drift progresses and $\dot{V}$O_2max_ decreases during steady-state exercise, the relative intensity of exercise for a given absolute workload represents a greater percentage of $\dot{V}$O_2max_, and therefore results in greater physiological and perceptual strain.

The National Institute for Occupational Safety and Health (NIOSH) outlines acceptable work standards for hot conditions [8]. They recommend an intensity of continuous work to be no more than 30% to 40% $\dot{V}$O_2max_ without the presence of environmental heat stress [8]. While performing at maximal capacity may not be required for many occupations, progressive reductions in $\dot{V}$O_2max_—and consequently increases in %$\dot{V}$O_2max_—that accompany cardiovascular drift could result in submaximal work intensities that exceed this recommended range, which may also negatively impact worker productivity and safety.

One heat stress mitigation strategy recommended by NIOSH is the incorporation of work-to-rest ratios. However, their efficacy with regards to blunting cardiovascular drift and concomitant reductions in $\dot{V}$O_2max_ has not been investigated. Most prior studies that examined cardiovascular drift and subsequent effects on $\dot{V}$O_2max_ utilized continuous work protocols [4,7,9,10], and those that incorporated rest did not measure $\dot{V}$O_2max_ [11] or utilized a prolonged 90 min rest period prior to the maximal exercise test [12].

The minimum work:rest ratio recommended by NIOSH is 45:15 min [8] and there is evidence that 15 min of rest is not adequate for preventing increases in thermal and cardiovascular strain [13]; if elevated body temperature persists, then some amount of ‘accumulated’ cardiovascular drift would be expected over consecutive work:rest cycles, which could result in reduced $\dot{V}$O_2max_. Without an understanding of the extent to which work capacity is reduced, optimizing work:rest ratios designed to mitigate physiological strain during work under heat stress is an improbable task. The purpose of this study was to test the hypothesis that during moderate-intensity work utilizing the recommend 45:15 min work:rest ratio, cardiovascular drift will accumulate over time. Furthermore, it was hypothesized that the magnitude of accumulated cardiovascular drift would be proportional to decrements in $\dot{V}$O_2max_.

## 2. Materials and Methods

A repeated measures crossover research design was utilized. Participants completed three sessions on different days, each separated by at least 48 h and not more than 1 week. The initial visit was for measurement of $\dot{V}$O_2max_ and familiarization. The remaining two experimental trials were counterbalanced, and the treatment orders were randomly assigned. For one experimental trial, participants performed simulated work at a moderate intensity for 120 min as two 45 min:15 min work:rest cycles. On a different day, participants performed simulated work for 15 min, immediately followed by measurement of $\dot{V}$O_2max_. The purpose of the separate 15 and 120 min trials was so that $\dot{V}$O_2max_ could be assessed before and after cardiovascular drift occurred since $\dot{V}$O_2max_ could not be assessed twice within the same 120 min trial. Other than duration, all aspects of the 15 and 120 min trials were identical.

All sessions occurred in a climate-controlled environmental chamber at approximately the same time of day for each participant to control for circadian variation in core temperature, measured as gastrointestinal temperature (T_gi_). Each female participant completed all experimental visits within a single phase of the menstrual cycle to control for fluctuations in baseline core body temperature; the menstrual cycle phase was not expected to influence study outcomes [14]. Resting T_gi_ was used to confirm self-reported menstrual cycle phase. Environmental conditions were established using indoor wet-bulb globe temperature (WBGT_in_) [15], and confirmed by a Kestrel 4400 Heat Stress Meter (Kestrel Meters, Boothwyn, PA). The initial visit occurred in temperate conditions [22 °C, 40% relative humidity (RH), WBGT_in_ = 17 °C], whereas remaining visits occurred under heat stress (34 °C, 55% RH, WBGT_in_ = 29 °C). The hot environmental conditions were chosen using data from the National Weather Service for the Southeastern United States intended to mimic the WBGT workers in this region experience during summer [16]. Air flow generated by the environmental chamber accounted for the NIOSH assumption of ‘perceptible air movement’ [8]. The 45:15 min work:rest ratio was determined from the NIOSH Recommended Alert Limits and the adjusted ambient temperature for the test environment [8]. The assigned workload aimed to elicit 201–300 kcal·h^−1^ (metabolic rate [*Ṁ*] = 234–349 W), which is defined as moderate intensity by the American Conference of Governmental Industrial Hygienists (ACGIH) and NIOSH [8,17]. Workload was evaluated as a time-weighted average, and to achieve moderate intensity work, participants performed arm curls, treadmill walking, and rest; arm curls were included in the protocol to vary the metabolic demand during simulated work [18]. Arm curls were completed while standing, using a total weight of 4.5 kg (2.25 kg in each hand) at a rate of 20 curls per min in time with a metronome. Treadmill walking was prescribed to achieve $\dot{V}$O_2_ = 1.0–1.1 L·min^−1^ at 4.0 km·h^−1^, and the grade was adjusted accordingly.

An a priori power analysis revealed eight participants were needed to observe a meaningful effect of cardiovascular drift on $\dot{V}$O_2max_, based on means, SDs, and an effect size from a prior study [4] using G*Power 3.1.9.4 [19]. To account for the individual characteristic assumptions of the NIOSH heat stress guidelines, participant inclusion criteria were healthy men and women who were “physically fit, well rested, fully hydrated, [and] under age 40” [8]. NIOSH does not define ‘physically fit’; this criterion was considered met if participants self-reported meeting the public health recommendation of ≥150 min of moderate-intensity physical activity per week or the equivalent [20]. Additional exclusion criteria included a history of metabolic, renal, or cardiovascular disease, gastrointestinal issues that contraindicate the use of an ingestible thermistor, or contraindications to exercise in the heat. Participants were instructed to arrive for all trials hydrated, rested, and having refrained from eating 2 h prior to the trial, as well as having avoided the use of alcohol, tobacco, and caffeine 24 h prior to testing.

*Initial Visit.* Written informed consent was obtained prior to participation. Hydration status was assessed via urine specific gravity (USG), with USG ≤ 1.020 considered adequately hydrated [21]. Percent body fat was estimated using the sum of skinfolds at three sites [22]. Height was measured using a stadiometer (model no. 213 1821009, Seca, Chino, CA, USA) and body mass was measured using a digital scale (BWB-800, Tanita Corporation, Tokyo, Japan) while the participant was wearing shorts, socks, sports bra (females), and tank top. Next, participants donned trousers (50% polyester, 50% cotton) and a long-sleeved buttoned shirt (65% polyester, 35% cotton), and were instrumented with a HR monitor around their chest (H10, Polar USA, Bethpage, NY, USA). Finally, participants were familiarized with the questions and scales for rating of perceived exertion (RPE) and rating of thermal sensation (RTS) [23,24].

A graded exercise test (GXT) was administered on a treadmill to measure $\dot{V}$O_2max_ (TrueOne 2400, Parvo Medics, Salt Lake City, UT, USA). Participants ran at a constant self-selected speed and an initial grade of 2.5%. The grade increased every 2 min by 2.5% until volitional exhaustion. RPE and HR were recorded 15 s before the end of each stage and at maximal exertion. Three minutes after the end of the test, a 2 mL blood sample was drawn from a superficial forearm vein or obtained via fingerstick to assess blood lactate (YSI 2300 STAT PLUS, YSI Inc., Yellow Springs, OH, USA; Lactate Plus Meter, Nova Biomedical, Waltham, MA, USA). After the GXT, participants rested for 20 min before completing a ~2 min $\dot{V}$O_2max_ plateau verification protocol during which they ran to volitional exhaustion [25]. The workload for the verification was determined by the last stage of the GXT: if it lasted <1 min, verification occurred at the same workload, but if it was ≥1 min, it occurred at the next stage (+2.5% grade). Lastly, the exercise intensity to be used during the experimental trials was verified and participants were familiarized with the CO_2_-rebreathing technique used to non-invasively determine cardiac output during exercise [26].

*Experimental Trials.* An ingestible thermometer pill (eCelsius Performance, BodyCap Medical, Hérouville-Saint-Clair, France), for the measurement of T_gi_, was provided for ingestion 5 h prior to arrival—6 h prior to the start of exercise—to ensure passage into the gastrointestinal tract and to avoid artifact by water consumption [27]. USG and nude body mass were assessed before exercise began, and participants again donned the same trousers and a long-sleeved shirt. Skin temperature was measured at four sites using wireless data loggers (iButton model no. DS1921H, Embedded Data Systems, Lawrenceburg, KY). Mean skin temperature ($\bar{T}$_sk_) was calculated as $\bar{T}$_sk_ = 0.3(*T*_1_ + *T*_2_) + 0.2(*T*_3_ + *T*_4_), where *T*_1_, *T*_2_, *T*_3_, and *T*_4_ correspond to skin temperature of the chest, lateral deltoid, quadriceps, and gastrocnemius, respectively [28]. T_gi_ and $\bar{T}$_sk_ were used to calculate weighted mean body temperature ($\bar{T}$_b_) as $\bar{T}$_b_ = 0.8(T_gi_) + 0.2($\bar{T}$_sk_) [29].

Next, two 2 mL blood samples were obtained from a superficial forearm vein for the baseline assessment of plasma osmolality, blood lactate concentration, hematocrit, and hemoglobin concentration. Whole blood was centrifuged at 3500× *g* for 15 min and plasma osmolality was measured using freezing point depression (Fiske Micro-Osmometer, Model 210, Advanced Instruments, Norwood, MA, USA). Hematocrit and hemoglobin concentration were used to calculate changes in plasma volume [30]. Participants then entered an environmental chamber maintained at a WBGT_in_ of 29 °C and sat quietly in a chair for 20 min to allow for equilibration to the hot environment and for preparation.

**Figure 1:**
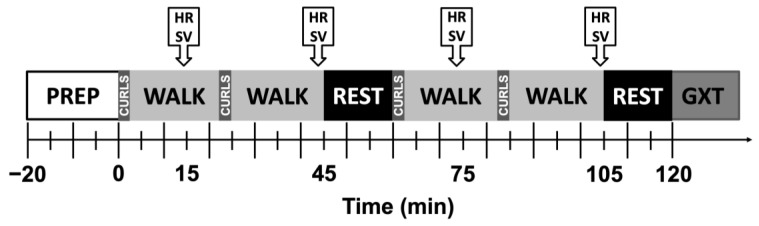
Procedures during the 120 min visit. Simulated work was achieved by 2.5 min of arm curls and 20 min of treadmill walking, repeated for a total of 45 min of work and followed by 15 min of rest. All baseline measurements were obtained before minute 0 except for hematological measures, which were taken before the participant entered the environmental chamber at minute −20. HR, heart rate; SV, stroke volume; GXT, graded exercise test.

Figure 1 illustrates procedures for the 120 min protocol (120MIN). Around minute 15 and just before minute 45 of each exercise bout, $\dot{V}$O_2_, RPE, RTS, and cardiac output were measured; SV was calculated from HR and cardiac output measures. T_gi_, HR, and skin temperature were recorded continuously. Participants remained in the environmental chamber, seated, for each rest break. At minute 120, participants immediately began a GXT with progressively increasing workloads in stages using the same speed and increases in grade (+2.5%) as the initial visit, running until volitional exhaustion to determine $\dot{V}$O_2max_. Three to 5 min after the GXT, a final blood sample was obtained via venipuncture to assess changes in blood lactate concentration, plasma osmolality, and plasma volume from baseline. Participants were allowed to drink cool water (~20 °C) ad libitum throughout, provided it did not impede the collection of data or completion of the protocol. Water temperature was determined as the mean temperature of 0, 60, and 120 min. Cardiovascular drift was assessed as changes in HR and SV between 15 min and 45, 75, and 105 min.

On a different day, participants completed a 15 min trial (15MIN) identical to the first 15 min of 120MIN; $\dot{V}$O_2max_ was measured during a GXT that began at minute 15. HR, T_gi_, and $\dot{V}$O_2_ were recorded during exercise. Like 120MIN, a blood sample was obtained ~3 min after the end of the test to assess blood lactate.

After exercise, participants were asked to towel off before measurement of nude body mass. Change in body mass was used in the calculation of whole body sweat loss, adjusted for fluid consumed, urine output, blood drawn, as well as estimated respiratory water loss and respiratory mass loss [31]. Total water consumption was the difference in pre- and post-mass of the participant’s water bottle.

Metabolic rate (*Ṁ*) during submaximal exercise was calculated from measures of $\dot{V}$O_2_ and RER [32]. Net metabolic heat production (*Ṁ*-*$\dot{W}$*) was calculated as the difference between *Ṁ* and external work rate (*$\dot{W}$)*. *Ṁ* and *Ṁ*-*$\dot{W}$* were calculated separately for arm curls, walking, and rest, and are reported as a time-weighted average per NIOSH recommendations [8].

Statistical analyses were performed using SPSS for Windows v.25 (IBM Corporation, Somers, NY, USA). Paired samples *t*-tests were used to test the significance of mean differences in $\dot{V}$O_2max_ between 15MIN and 120MIN, as well as hematological variables pre- and post-exercise. For continuous variables such as cardiovascular, thermal, and metabolic measures, a one-way repeated measures analysis of variance (ANOVA) was conducted to compare values across time. If sphericity was violated, the Greenhouse-Geisser correction was applied. In the event of a significant omnibus test, pairwise comparisons with a Bonferroni α correction were performed. For ordinal variables (RPE; RTS), a nonparametric Friedman’s ANOVA was used. All statistical tests used an α level of 0.05.

## 3. Results

Eight participants (five women) completed all study procedures (mean ± SD; age = 25 ± 5 y [range: 18–35], body mass = 74.8 ± 11.6 kg, height = 1.73 ± 0.10 m, % body fat = 24.0% ± 9.0%, $\dot{V}$O_2max_ = 42.9 ± 5.6 mL·kg^−1^·min^−1^). Environmental conditions for all visits were achieved as intended (Table 1).

### 3.1. Responses during Submaximal Exercise and Rest Intervals

Participants were adequately hydrated at the beginning of all visits (USG: INITIAL = 1.008 ± 0.003; 15MIN = 1.007 ± 0.007; 120MIN = 1.009 ± 0.005). Plasma osmolality at baseline was 291 ± 5 mOsm·kg^−1^ and increased 5% to 305 ± 10 mOsm·kg^−1^ (*p* = 0.002) post-GXT, which is likely in part due to fluid movement out of the vasculature, with maximal exercise [33] reflected in the 7.5% ± 3.1% decrease in plasma volume from baseline to the end of exercise (*p* < 0.001). During 120MIN, participants consumed 0.44 ± 0.30 L of water (21.4 ± 4.4 °C) ad libitum. Water consumption did not fully replace sweat losses of 1.2 ± 0.1 L (*p* = 0.001), resulting in a mean body mass loss of 0.9% ± 0.4%.

$\dot{V}$O_2_ was maintained at an average of 1.08 ± 0.05 L·min^−1^ (35.8% ± 9.1% $\dot{V}$O_2max_) during treadmill walking (*p* < 0.001), but despite statistical significance, mean differences across all time points were not greater than 0.05 L·min^−1^ (Table 2A). $\dot{V}$O_2_ was 0.54 ± 0.08 L·min^−1^ (17.5% ± 3.2% $\dot{V}$O_2max_) during arm curls. RPE also exhibited an upward drift over time (*p* = 0.003; Table 2A).

As intended, all participants’ work intensity was categorized as moderate based on NIOSH guidelines [8] when work intensity was expressed as kcal·h^−1^ (240 ± 13 [range: 224–262]) and calculated as *Ṁ* (279 ± 15 W [range: 260–304 W]). Accounting for external work, *Ṁ*-*$\dot{W}$* was 231 ± 30 W (range: 204–288 W).

**Figure 2:**
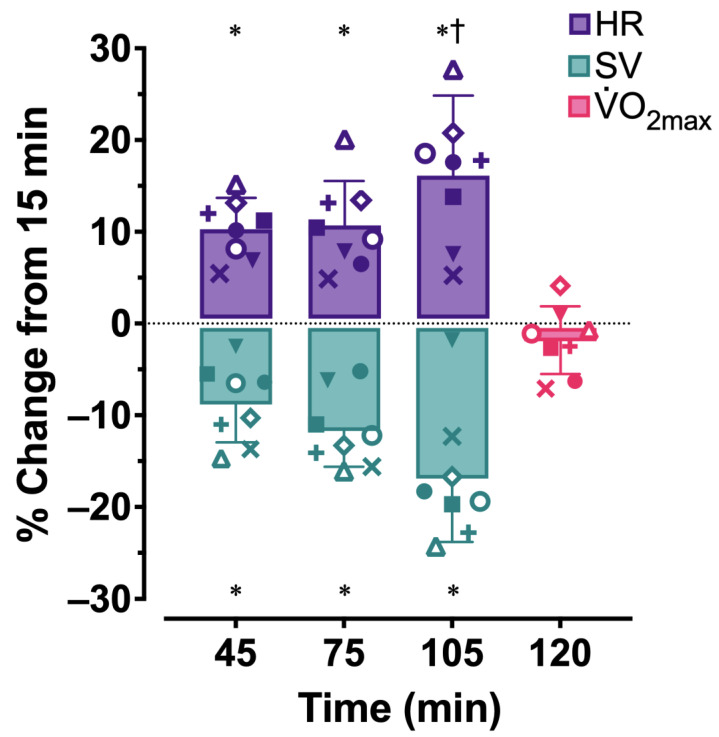
Individual and mean ± SD percent change from minute 15 for heart rate (HR), stroke volume (SV), and maximal oxygen uptake ($\dot{V}$O_2max_). Bars represent group means and symbols correspond to responses for each participant. * *p* < 0.05 compared to 15 min; † *p* < 0.05 compared to 75 min.

Cardiovascular drift occurred as evidenced by a 16.7% increase in HR (*p* < 0.001) and a 16.9% decrease in SV (*p* < 0.001) from 15 to 105 min, and in fact, cardiovascular drift accumulated over consecutive work bouts (Table 2A and Figure 2). HR rose from 15 to 45 min (*p* = 0.001), was not different between 45 and 75 min (*p* = 1.00), and increased further from 75 to 105 min (*p* = 0.04). SV decreased from 15 to 45 min (*p* = 0.002), remained steady (*p* = 0.09) between 45 and 75 min, and, despite an additional 6.5% reduction, was not statistically different (*p* = 0.37) between 75 and 105 min.

HR during arm curls—the final minute of each set (at 2, 24, 62, and 84 min)—also exhibited accumulated drift of +28.8% (*p* < 0.001), increasing from set 1 (104 ± 13 beats·min^−1^) to set 2 (123 ± 17 beats·min^−1^; *p* = 0.003), remaining fairly stable between sets 2 and 3 (118 ± 15 beats·min^−1^; *p* = 0.30), and rising again from set 3 to 4 (134 ± 16 beats·min^−1^; *p* = 0.03). Cardiac output measurement was not feasible during arm curls, so SV data are unavailable for these time points. Rest intervals were inadequate for HR to return to baseline as evidenced by elevated HR at the end of both the first (*p* = 0.004) and second (*p* < 0.001) rest breaks (*p* < 0.001; Table 2B).

**Figure 3:**
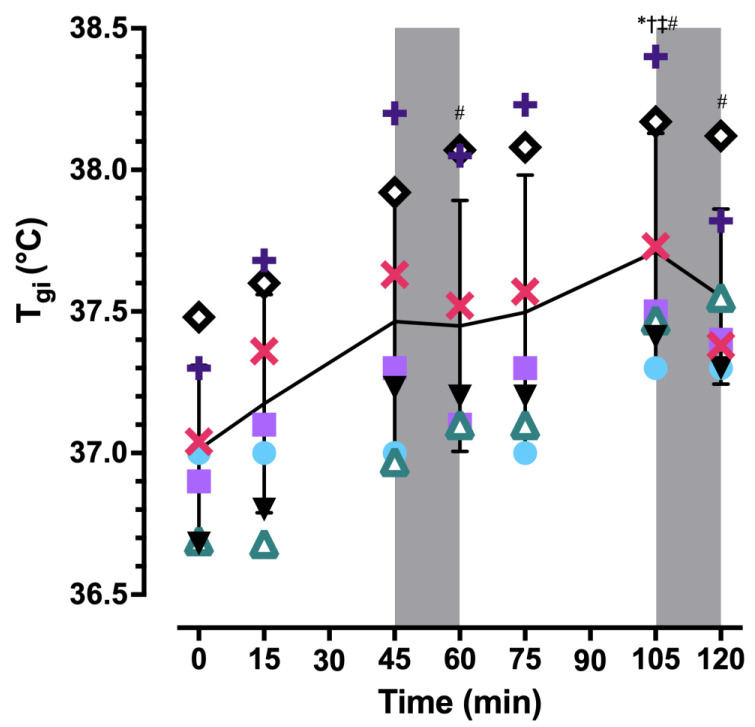
Individual and mean ± SD gastrointestinal temperature (T_gi_) over two 45:15 min work:rest cycles. The solid line corresponds to mean T_gi_ and the symbols correspond to responses for each participant. Shaded areas represent rest breaks. N = 7 at all time points because of technical difficulties for one participant that resulted in missing data at 45, 75, and 105 min. * *p* < 0.05 compared to 15 min; † *p* < 0.05 compared to 45 min; ‡ *p* < 0.05 compared to 75 min; # *p* < 0.05 compared to baseline (0 min).

**Figure 4:**
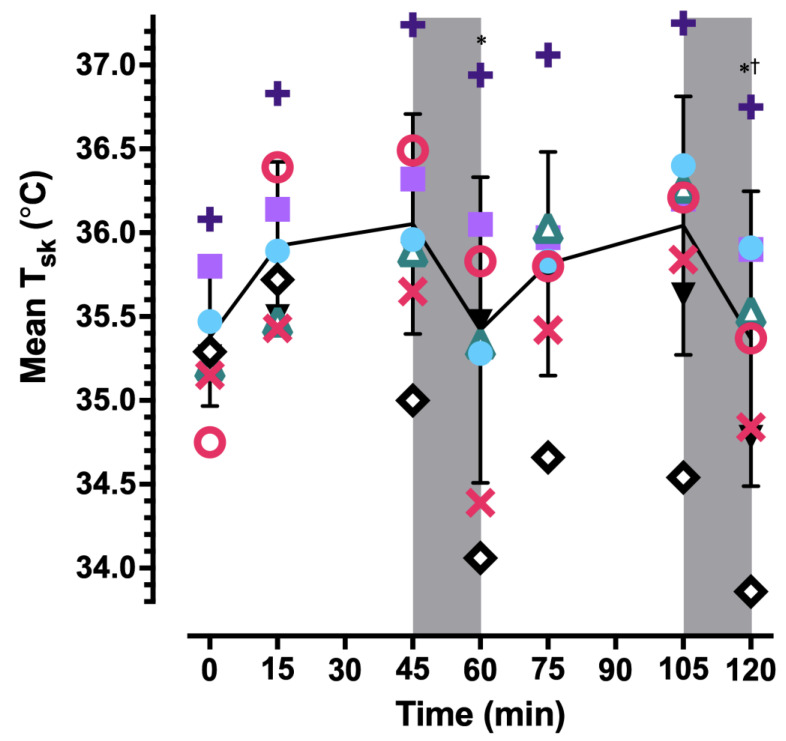
Individual and mean ± SD mean skin temperature (T_sk_) over two 45:15 min work:rest cycles. The solid line corresponds to the average mean T_sk_ for the entire sample and the symbols correspond to responses for each participant. Shaded areas represent rest breaks. * *p* < 0.05 compared to 45 min. † *p* < 0.05 compared to 105 min.

T_gi_ data were not available at all timepoints for one participant due to intermittent loss of communication with the monitor; these data were excluded from statistical analyses. At 120 min, T_gi_ (N = 7) was elevated 0.5 ± 0.2 °C from baseline (*p* = 0.006; Figure 3). Neither rest break was sufficient to decrease T_gi_ from the end of the preceding work bout (both *p* = 1.00). $\bar{T}$_sk_ was elevated at 45 min compared to the end of the first (*p* = 0.02) and second (*p* = 0.047) rest breaks but not compared to baseline (*p* = 0.29; Figure 4). $\bar{T}$_sk_ was also elevated at 105 min compared to the end of the rest break that immediately followed (*p* = 0.002). Over 120 min, the core-to-skin temperature gradient (T_gi_−$\bar{T}$_sk_) gradually increased (Table 2A,B) because of a slowly rising T_gi_ and the return of $\bar{T}$_sk_ to baseline during each rest break (*p* = 0.02). $\bar{T}$_b_ exhibited a similar slow rise over time (*p* < 0.001; Table 2A,B).

While RTS did not remain the same throughout the work bouts of 120MIN, post hoc comparisons showed that RTS was not significantly greater at any timepoint when compared to 15 min (Table 2A). However, participants felt hotter during rest compared to baseline: RTS remained elevated at both 60 min (*p* = 0.04) and 120 min (*p* = 0.006; Table 2B).

### 3.2. Responses to Maximal Exercise

Despite an accumulated increase in HR and decrease in SV over time, $\dot{V}$O_2max_ was not different between 15 min and 120 min (*p* = 0.17; Figure 2 and Table 3). Along with absolute and relative $\dot{V}$O_2_, RPE, RTS, and blood lactate responses were not different among maximal exercise bouts (all *p* > 0.05). Average maximal HR achieved during 120MIN was 1% lower than that during the initial visit (*p* = 0.01; Table 3); all participants achieved a maximal HR within 5 bpm of the initial GXT. With the exception of one participant, the final workload (treadmill grade and speed) during each GXT for each participant was identical.

While T_gi_ (N = 7) increased over time during 120MIN, the rest break just prior to the GXT was sufficient to render T_gi_ at the end of the GXT comparable (*p* = 0.25) to T_gi_ at the end of the GXT in 15MIN (Table 3). The longer running duration during the 15MIN GXT (Table 3) might have also contributed to a T_gi_ comparable to that in 120MIN despite the shorter submaximal exercise period before measurement of $\dot{V}$O_2max_ during 15MIN.

## 4. Discussion

The purpose of this study was to determine the extent to which cardiovascular drift ‘accumulated’ over consecutive work:rest cycles and the subsequent impact on work capacity, indexed as $\dot{V}$O_2max_. As hypothesized, the primary finding was that cardiovascular drift accumulated over multiple work bouts; however, $\dot{V}$O_2max_ was unaffected. The statistically nonsignificant 2% decrease in $\dot{V}$O_2max_ after two work:rest cycles in 120MIN compared to 15MIN corresponded to comparable GXT durations and final workloads in the majority of participants. The observed change in $\dot{V}$O_2max_, as well as the magnitude of all individual responses, was within the expected day-to-day variability for repeated $\dot{V}$O_2max_ testing [34].

The magnitude of cardiovascular drift observed in the current study was comparable to other studies involving prolonged exercise in the heat. Gliner et al. [11] observed responses over 4 h of 50:10 work:rest at 35% $\dot{V}$O_2max_ in the heat (35 °C, 30% RH), and found similar magnitudes of drift in HR and SV—+23% and approximately −23%, respectively—but $\dot{V}$O_2max_ was not assessed at the conclusion of the protocol. Other studies observed a comparable magnitude of cardiovascular drift of 9–19% and a concomitant decrement in $\dot{V}$O_2max_ [4,7,9,10,35], but despite the similar magnitude of cardiovascular responses, $\dot{V}$O_2max_ was unaffected in the current study.

Contrary to other studies demonstrating that the elevated HR associated with cardiovascular drift reflects an increased relative metabolic intensity as a result of accompanying decreases in $\dot{V}$O_2max_ [4,10,35], the increase in HR in the present study appeared to be dissociated from relative metabolic intensity since $\dot{V}$O_2max_ was preserved at the end of two work:rest cycles. Despite this, RPE increased slightly over consecutive work bouts, which is consistent with the observed increase in HR that is associated with cardiovascular drift. However, the precise explanation for this result is elusive since we did not observe a change in relative metabolic intensity (i.e., %$\dot{V}$O_2max_). The ~0.5 °C increase in T_gi_ between 15 and 105 min may have contributed, as RPE has been shown to be amplified by elevated body temperature [36,37].

Although the relative magnitudes of drift (i.e., % change) in HR and SV in the present study were comparable to the studies cited above, the absolute values for HR and SV were lower in this study. The absolute and relative work intensities utilized in this study were lower, which resulted in a lower absolute metabolic demand and elicited HR of 110–130 beats·min^−1^ during exercise, compared to 150–170 beats·min^−1^ in others [4,7,9,10,35]. This difference in working HR resulted in a greater cardiac reserve (i.e., the difference between exercise values and maximum values) in the present study. The rest period prior to measurement of $\dot{V}$O_2max_ in this study also may have facilitated greater cardiac reserve considering the other cited studies did not include a rest period between submaximal exercise and measurement of $\dot{V}$O_2max_. It may be that there is a minimum threshold work intensity and accompanying cardiovascular drift involving higher absolute HR and SV, and therefore greater reductions in cardiac reserve—rather than a given magnitude of cardiovascular drift per se—that is most strongly associated with reductions in $\dot{V}$O_2max_ during prolonged work in the heat.

Given that previous studies observed a decrease in $\dot{V}$O_2max_ preceded by a similar magnitude of cardiovascular drift, why was $\dot{V}$O_2max_ unaffected in the current study after 120 min? The data collected do not permit a definitive answer to this question, but preservation of SV during maximal exertion is the likely explanation. Compared to other studies [4,7,9,35], this study utilized a lower exercise intensity, longer duration, and inclusion of rest breaks. As a result, *Ṁ*-*$\dot{W}$* was 2–3 times greater in the other studies than the 231 W observed here. Higher *Ṁ*-*$\dot{W}$* caused greater increases in core temperature (even over shorter durations) that exacerbated cardiovascular strain—i.e., reduced SV—during submaximal exercise that likely persisted during maximal exercise. In contrast, despite a similar magnitude of cardiovascular drift in the current study, thermal strain was mild. This may have resulted in preservation of SV—and thereby maximal cardiac output—during maximal exercise. This notion is supported by findings from Saltin and Stenberg [38] who observed a comparable magnitude of cardiovascular drift—increase in HR and decrease in SV of ~15% each—during prolonged, intermittent, submaximal exercise (70% $\dot{V}$O_2max_, 195 min), but only a small reduction (5%; 0.1 L·min^−1^) in $\dot{V}$O_2max_ after a 90 min rest period. Despite the magnitude of decrease in SV during submaximal exercise, it was restored during maximal exercise, essentially preserving $\dot{V}$O_2max_. The authors attributed the ability to achieve maximal SV, and thereby maximal cardiac output, to a reduced skin blood flow requirement during the rest prior to measurement of $\dot{V}$O_2max_ [35]. It may be that a rest period shorter than 90 min, such as the 15 min utilized in the current study, is sufficient in reducing skin blood flow requirements under conditions of relatively low exercise intensity and thermal strain. While we were unable to measure skin blood flow, the return of $\bar{T}$_sk_ to baseline values before the GXT to measure $\dot{V}$O_2max_ is suggestive of reductions in skin blood flow during rest [39]. Since maximal HR was achieved and because (a-$\bar{v}$)O_2_ difference would not be expected to be different under these conditions [40], restoration of maximal SV by reductions in skin blood flow would have permitted achievement of maximal cardiac output and thereby $\dot{V}$O_2max_.

One limitation of this study is that we were not able to measure skin blood flow because of constraints regarding the instrumentation and the movement artifact associated with exercise, and so we can only speculate regarding the redistribution of blood flow to the skin. While skin temperature can be an adequate substitution for measurement of skin blood flow, participants wore a long-sleeved shirt and pants, creating a microenvironment that could have artificially elevated skin temperatures and overestimated the assumption of elevated skin blood flow; however, because skin temperature returned to baseline values during rest, we do not believe that to be the case.

Although NIOSH assumptions (age, physical fitness, health status, hydration, sleep, work intensity, environmental conditions) were accounted for in subject recruitment and study design, participants were likely not representative of the average worker, and so the extent to which these findings are applicable in the workplace is limited by the sample population. By design, participants did not have any co-morbidities, were <40 years old, and arrived at all laboratory visits prepared for peak performance: well rested, hydrated, and having refrained from recent alcohol or caffeine consumption. These ideal conditions are likely a less frequent reality, and as such generalization of these results to what workers experience warrants caution.

Even though assumptions of the ideal worker were largely met, several NIOSH recommendations were not fulfilled. Ad libitum water consumption did not meet the NIOSH-recommended rate of consumption of 0.95 L·h^−1^. NIOSH also recommends that work intensity does not exceed 30–40% $\dot{V}$O_2max_. $\dot{V}$O_2_ for two participants exceeded the upper end of this recommended range (>40% $\dot{V}$O_2max_), while three participants’ relative workloads remained below 30% $\dot{V}$O_2max_ and three participants’ relative workloads were between 30–40% $\dot{V}$O_2max_, although mean $\dot{V}$O_2_ remained within the prescribed range for all participants throughout the entirety of the protocol. In addition, two participants exceeded the NIOSH recommendation for core body temperature during work (≥38.0 °C) during the protocol.

Because of time constraints related to study execution, data were collected between October and January, and the NIOSH assumption that workers are heat acclimatized was left unmet/unconfirmed. However, this should have a limited impact, if any, on the findings. Two trials conducted in the heat would not likely have induced any degree of acclimation, and counterbalanced treatment orders should have eliminated any systematic acclimation effect on results if there had been one. Heat acclimation alters physiological set points: for a given submaximal exercise intensity, HR, core body temperature, skin temperature, and perceived exertion are lower, while sweat rate is higher, due to changes in the homeostatic mechanisms through which they function [41]. So, while absolute values might have been different if participants had been heat acclimated, the nature of the responses between 15 and 120 min should not have been affected.

Future research should expand upon the relationship between cardiovascular drift and $\dot{V}$O_2max_ at varying work intensities, environmental conditions, clothing requirements, work:rest ratios, and over longer durations. The next steps should include direct assessment of changes in skin blood flow to better understand the extent to which blood flow demand impacts cardiac reserve and $\dot{V}$O_2max_. Additionally, the exploration of cardiovascular drift in a sample population that is more representative of the actual working population is recommended, since it is unknown whether co-morbid conditions impact the relationship between cardiovascular drift and $\dot{V}$O_2max_.

## 5. Conclusions

This study examined the relationship between work intensity, cardiovascular drift, and $\dot{V}$O_2max_ over 120 min of intermittent exercise in the heat. During simulated moderate intensity work using NIOSH-recommended 45:15 min work:rest ratios in a hot environment, cardiovascular drift accumulated, resulting in a +17% and −17% change in HR and SV, respectively, compared to 15 min. T_gi_ also rose by an average of 0.5 °C over 2 h. Despite elevations in both cardiovascular and thermal strain persisting over consecutive 45 min work bouts coupled with 15 min rest periods, $\dot{V}$O_2max_ was unaffected. The 15 min rest periods were not sufficient to fully return HR or T_gi_ to initial resting values, but they were sufficient to preserve (or restore) work capacity when indexed as $\dot{V}$O_2max_.

## Figures and Tables

**Table 1:** Environmental conditions for all visits (mean ± SD). The 15 min (15MIN) and 120 min (120MIN) experimental trials were not different for any of the conditions.

Trial	Dry-Bulb Temperature (°C)	Relative Humidity (%)	WBGT_in_ (°C)	Wind Speed (m·s^−1^)
INITIAL	23.1 ± 0.8	44.3 ± 10.4	18.1 ± 1.2	0.4 ± 0.6
15MIN	34.5 ± 0.6	54.3 ± 3.4	29.0 ± 0.7	1.4 ± 0.1
120MIN	34.3 ± 0.4	55.9 ± 2.5	29.0 ± 0.4	1.4 ± 0.1

WBGT_in_, indoor wet-bulb globe temperature.

**Table 2:** Responses during the 120-min work:rest protocol.

A. Responses during treadmill walking (mean ± SD)				
	15 min	45 min	75 min	105 min
$\dot{V}$O_2_ (L·min^−1^) **	1.05 ± 0.05	1.08 ± 0.04 *	1.07 ± 0.05	1.10 ± 0.06 *^‡^
%INITIAL $\dot{V}$O_2max_ **	34.9 ± 9.2	36.1 ± 9.3 *	35.7 ± 8.8	36.5 ± 9.2 *^‡^
$\dot{Q}$ (L·min^−1^) **	7.8 ± 0.6	7.9 ± 0.6	7.6 ± 0.6	7.5 ± 0.6
HR (beats·min^−1^) **	110 ± 22	122 ± 24 *	122 ± 24 *	128 ± 26 *^‡^
SV (mL) **	73.1 ± 14.2	66.9 ± 15.1 *	64.9 ± 15.5 *	60.7 ± 13.3 *
T_gi_−$\bar{T}$_sk_ (°C)	1.3 ± 0.4	1.5 ± 0.7	1.7 ± 0.8	1.7 ± 0.9
$\bar{T}$_b_ (°C) **	36.9 ± 0.4	37.2 ± 0.4 *	37.2 ± 0.4	37.4 ± 0.4 *^†‡^
RPE **	10 ± 2	12 ± 2	11 ± 2	12 ± 3 *
RTS **	5.5 ± 0.5	6.0 ± 0.5	6.0 ± 0.5	6.0 ± 0.5
B. Responses during the final minute of rest intervals (mean ± SD)				
	Baseline	60 min	120 min	
HR (beats·min^−1^) **	68 ± 10	83 ± 11 ^§^	91 ± 15 ^§^^	
T_gi_−$\bar{T}$_sk_ (°C) **	1.6 ± 0.4	2.1 ± 1.1	2.2 ± 1.1	
$\bar{T}$_b_ (°C) **	36.7 ± 0.3	37.0 ± 0.4 ^§^	37.1 ± 0.3 ^§^	
RTS **	4.6 ± 0.3	5.4 ± 0.6 ^§^	5.4 ± 0.6 ^§^	

A. $\dot{V}$O_2_, oxygen uptake; %INITIAL $\dot{V}$O_2max_, as a percentage of $\dot{V}$O_2max_ as measured during the initial visit; $\dot{Q}$, cardiac output; HR, heart rate; SV, stroke volume; T_gi_−$\bar{T}$_sk_, gastrointestinal-to-skin temperature gradient; $\bar{T}$_b,_ mean body temperature; RPE, rating of perceived exertion; RTS, rating of thermal sensation. For T_gi_−$\bar{T}$_sk_ and $\bar{T}$_b_, N = 7 at all time points because of technical difficulties for 1 participant resulting in data missing at 45, 75, and 105 min. For all other variables, N = 8. * *p* < 0.05 compared to 15 min; † *p* < 0.05 compared to 45 min; ‡ *p* < 0.05 compared to 75 min; ** *p* < 0.05 for the omnibus test. B. HR, heart rate; T_gi_−$\bar{T}$_sk_, gastrointestinal-to-skin temperature gradient; $\bar{T}$_b,_ mean body temperature. N = 7 at all time points because of technical difficulties for 1 participant that resulted in missing data. § *p* < 0.05 compared to baseline; ^ *p* < 0.05 compared to 60 min; ** *p* < 0.05 for the omnibus test.

**Table 3:** Responses to maximal exercise (mean ± SD).

	Initial	15MIN	120MIN
$\dot{V}$O_2_ (L·min^−1^)	3.20 ± 0.90	3.22 ± 0.95	3.16 ± 0.92
$\dot{V}$O_2_ (mL·kg^−1^·min^−1^)	42.0 ± 5.7	42.3 ± 6.3	41.5 ± 6.1
RER^**^	1.16 ± 0.06	1.12 ± 0.07	1.10 ± 0.08 *
HR (beats·min^−1^) **	191 ± 5	188 ± 6	189 ± 5 *
T_gi_ (°C)	–	37.6 ± 0.3	37.7 ± 0.3
RPE	19 ± 1	19 ± 1	19 ± 1
RTS	–	7.0 ± 0.5	7.0 ± 0.5
Blood lactate (mmol·L^−1^)	6.3 ± 1.4	5.9 ± 2.0	6.0 ± 3.1
Treadmill grade (%)	11.1 ± 1.5	11.1 ± 2.8	10.8 ± 2.7
Speed (km·h^−1^)	8.2 ± 0.8	8.3 ± 0.8	8.3 ± 0.8
GXT duration (s) **	480 ± 66	400 ± 66	321 ± 88 *^†^

INITIAL, initial visit; 15MIN, 15 min protocol; 120MIN, 120 min protocol; $\dot{V}$O_2_, oxygen uptake; RER, respiratory exchange ratio; HR, heart rate; T_gi_, gastrointestinal temperature; RPE, rating of perceived exertion; RTS, rating of thermal sensation; GXT, graded exercise test. Data for T_gi_ are based on N = 7; data for all other variables are based on N = 8. Three blood lactate measures were taken from a fingerstick blood sample (one each for INITIAL, 15MIN, 120MIN; three different participants) due to venipuncture or equipment difficulties. T_gi_ and RTS were not collected during the initial visit; RPE and RTS were analyzed using nonparametric statistics. * *p* < 0.05 compared to INITIAL, † *p* < 0.05 compared to 15 min; ** *p* < 0.05 for the omnibus test.

## Data Availability

Data are available upon reasonable request from the corresponding author.
